# Adaptation of an intervention for psychological distress in children and adolescents living in urban areas of Sweden – UNITE

**DOI:** 10.3389/fpubh.2026.1870070

**Published:** 2026-08-10

**Authors:** Erica Mattelin, Josefin Michanek, Camila Demmou, Hanna Thermaenius, Sandra Tielman, Cina Rydergård, Sara Skoog, Aemal Akhtar

**Affiliations:** 1 Department of Clinical Neuroscience, Karolinska Institutet, Stockholm, Sweden; 2 Save the Children Sweden, Stockholm, Sweden; 3 Liverpool School of Tropical Medicine, Liverpool, United Kingdom

**Keywords:** children, mental health, Sweden, task-sharing, violence

## Abstract

**Background:**

Sweden has faced an increase in community violence over the past few years. The integration of potentially scalable psychological interventions into mental health responses in affected communites can contribute to addressing the psychological consequences. The World Health Organization's *Early Adolescence Skills for Emotions (EASE)* was originally developed as a task-sharing, group-based intervention for low- and middle-income countries. To address urgent needs in a high-income context, EASE was pragmatically adapted into a shorter, low-intensity intervention named UNITE, suitable for rapid implementation within civil society settings.

**Methods:**

UNITE was developed through a structured but pragmatic adaptation process, including contextual needs assessment, stakeholder consultation, cultural and linguistic modifications, and adjustments to format, intensity, and delivery structure. The intervention was shortened and modified to fit the operational realities of community-based delivery and to enable implementation by non-mental health specialists within the framework of Save the Children Sweden's regular activities in areas affected by community violence.

**Results:**

The adaptation process resulted in a more brief, scalable group intervention tailored to high-income urban contexts experiencing chronic community violence. Key adaptations included reduced session length, streamlined content, and alignment with existing organizational infrastructure to facilitate feasibility and sustainability. UNITE was successfully implemented within routine programming, demonstrating the practicality of adapting task-sharing psychological interventions across economic contexts. Lessons learned highlight the importance of balancing fidelity to core therapeutic components with contextual responsiveness.

**Conclusion:**

This study provides a real-world example of adapting a WHO-developed task-sharing intervention from a low- and middle-income setting to a high-income context. The findings underscore the potential of scalable, low-intensity interventions delivered by non-specialists in these contexts and offer guidance for civil society organizations seeking to implement rapid, evidence-informed mental health responses in similar settings.

## Background

Community violence is defined as deliberate acts intended to cause physical harm against a person or persons residing in the community (1). This form of violence, not only impacts those directly involved but also leaves a lasting mark on the children and youth growing up in these areas (2). In recent years, Sweden has witnessed a concerning rise in severe community violence, including shootings, bombings, and fatal and non-fatal stabbings (3, 4). During 2020–2022 nearly 700 shootings have occurred in close proximity to schools (5). Many of the areas that have been most exposed to these explicit forms of interpersonal violence are also socioeconomic deprived areas, where children live within a context of structural violence and are exposed to frequent stressors such as poverty, social problems and racism (6). Research from other countries, notably the United States, where community violence has a historical footprint, suggests that children residing in violent areas witness multiple forms, ranging from robberies and drug trafficking to more severe incidents like shootings or knife attacks (7).

Growing up amidst community violence has an impact on the mental health of children. When children witness violence or other frightening events, they can experience impacts on their mental wellbeing immediately. Common reactions can include sleep disturbances, event-related intrusive thoughts, and increased irritability, especially among teenagers (8). Most children recover from these acute symptoms within months of exposure (9, 10), but it has been estimated that approximately 16% go on to fulfill posttraumatic stress disorder (PTSD) diagnosis (11, 12). Meta-analyses reveal that community violence is associated with PTSD and internalizing symptoms such as anxiety and depressive symptoms in children (2). The most robust predictor of symptomatology is victimization, surpassing the impact of merely witnessing or hearing about it. Adolescents, compared to younger children, report a stronger relationship between externalizing behaviors and exposure, while children exhibit greater internalizing problems.

In addition to the increased risk of developing mental health problems, many feel unsupported by their society in coping with these challenges (13). The experience of staff that meet children directly is that when many children in Sweden need crisis support at the same time, the Swedish system does not function sufficiently. This became more prominent during the large immigration that took place in 2015, when many accompanied and unaccompanied children came to Sweden and their needs for support and treatment were not met (14). One of the area's most profoundly affected by community violence in Sweden is Järva (15). In this area, children not only now face a higher risk of exposure to community violence but also, to a greater extent, come from migrant and refugee backgrounds and may have previously escaped violence only to find themselves in a new environment marked by further violence and reduced access to child psychiatric care (16).

When children and youth experience traumatic events, immediate psychological first aid is recommended during the initial days (17, 18), followed by appropriate psychological treatment when needed. However, there is currently a lack of preventive interventions in the physical areas where many children experienced severe events. One existing evidence-based intervention for children living in adversity is Early Adolescence Skills for Emotions (EASE), developed by the World Health Organization (WHO) (19–21). EASE is a cognitive behavioral therapy (CBT) based intervention that has been carried out in groups of children affected by different forms of adversity. Interventions such as EASE are viable options for providing psychosocial care in such settings since they are implemented through *task sharing*, in which individuals without previous formal training in social or psychological work are taught a method and supervised to support children and parents (22). Task sharing has been recommended globally to aid in combatting the large mental health treatment gap that exists (23), as there is a global shortage of professionals who can provide mental health care (24). These methods have mainly been used in countries and areas with strained healthcare resources, such as humanitarian crises and in countries without well-developed mental health systems (25). In recent years, however, both the working method and the research have begun to move toward high-income countries such as the Netherlands and Switzerland (26, 27). Task sharing has been tested for different diagnoses, and transdiagnostically, with positive results (28–30).

However, EASE has not yet been adapted for a high-income setting and may potentially face hurdles when attempting to integrate into healthcare frameworks in these contexts. Furthermore, there is a gap in interventions to identify and guide children with a higher symptom burden toward the appropriate authorities during the period between these two types of interventions. Given the substantial number of children affected by community violence and the existing gap in interventions, urgent action is needed. One potential solution could be the implementation of a psychological intervention in affected areas, delivered by staff from civil society organizations with good contextual knowledge, but without prior experience in mental health care. Earlier studies have indicated that CBT-based interventions are effective for internalizing behaviors and health-care-seeking behavior when delivered in schools (31, 32). However, to our knowledge, this pilot represents the first attempt to establish the feasibility, safety, and acceptability of an adapted psychological intervention delivered by civil society to children affected by community violence in Sweden.

To guide the adaptation process, we used Bernal's Framework (33), which outlines eight dimensions relevant for cultural adaptation of psychological interventions: language, persons, metaphors, content, concepts, goals, methods, and context. The framework was chosen because it has been successfully applied in the adaptation of similar psychological interventions (34) and offered a practical and structured approach for the development of UNITE.

This paper describes the process of adapting a publicly available evidence-based potentially scalable psychological intervention to the Swedish context. The primary aim was to adapt the EASE intervention to allow for implementation and to adapt the contents to be relevant to children living in areas affected by community violence in Sweden.

## Method

### Setting

Sweden is a country with a population of 10 million people. In recent decades, an increasing number of so-called disadvantaged areas have emerged in the county. These areas are characterized by populations with low socioeconomic status and where criminal networks have a significant presence (35). Furthermore, there has been an increase in community violence with the consequence that more children are exposed to having witnessed or heard about violence and shootings or even being perpetrators or victims.

One of these vulnerable areas is Järva in Stockholm (35). Järva is an area with approximately 100,000 inhabitants that has been affected by shootings in recent years (36). Järva consists of several areas and three of them, Rinkeby, Husby and Tensta, are considered disadvantaged (35). Save the Children Sweden, a Swedish civil society organization, has been active in Järva since 2010. Currently, service provision primarily focuses on strengthening the engagement, participation and impact of children in the area, through the *On Equal Terms* program (37). For several years, it has been increasingly clear that there is a need for greater support for children in the area who have experienced community violence.

Additionally, Save the Children Sweden runs treatment centers for children that have experienced traumatic events. The unit consists of approximately 15 psychologists and psychotherapists with extensive experience of working with children in vulnerable life situations. The treatment centers are present in three locations: Stockholm, Gothenburg and Malmo. These treatment centers are not part of the national health care system but provides care for those children who have not been able to access care. As part of the long-standing work at the centers Save the Children Sweden has identified health care needs for children. One of the identified needs has been for the availability of task-sharing interventions in Sweden, with the hopes that such programs can eventually be integrated into the primary health care system.

Save the Children Sweden's already ongoing activities include staff who have extensive experience of working with children and young people and contextual competence, without formal mental health training. In Save the Children's activities, various types of psychosocial support are regularly underway. Some of these are based on CBT techniques and some more generic. Formal requirements for the staff involved in this project are only that they have completed upper secondary education, but the experience varies greatly in the group. These staff, as well as some recruited staff, were planned to provide the intervention after training and are hereinafter referred to as group leaders.

### Adaptation process

#### EASE

The Early Adolescent Skills for Emotions (EASE) manual is a potentially scalable intervention developed by the WHO (21). The active ingredients included are evidence-based and based on common CBT techniques. The intervention was developed to target symptoms of internalizing disorders in children (aged 10–15 years). Initial trials have indicated that EASE is successful in alleviating these symptoms, and a range of other symptoms, while simultaneously improving wellbeing (38, 39). To adapt the manual to the Swedish context, stakeholders indicated that a shorter intervention was needed to be able to scale implementation in the community and due to the already existing resources available in the national health-care system. Further, clinical psychologists specializing in child mental health emphasized the need for a low-threshold intervention that circumvents structural and systemic barriers limiting access to care, and does not require extensive screening prior to participation.

### Clinical workshops

**Figure 1:**
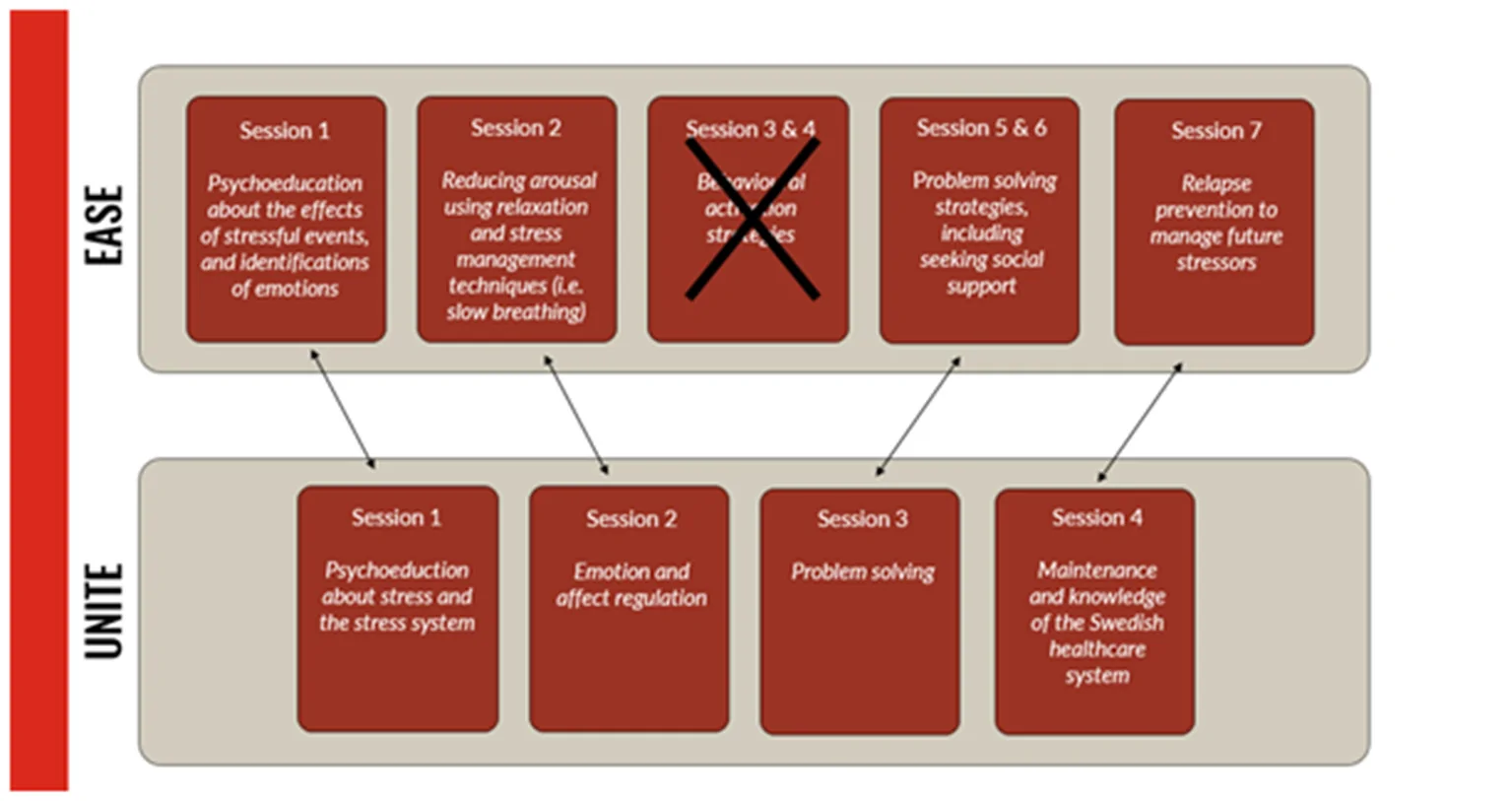
Adaptation of the original EASE intervention into the four-session UNITE intervention. Arrows indicate how content from the EASE sessions was retained and reorganized in UNITE.

The initial adaptation process began with four therapists going through the EASE implementation manual session by session (40) (Figure 1). This was done with the aim of identifying which adaptations needed to be made to the material linguistically, visually and content-wise. The four therapists were all employed at Save the Children Sweden. They had extensive experience working with children experiencing difficult life situations, with an average practice time of 16.5 years. Three of the therapists had prior experience in working with and supervising other task-sharing psychological interventions in Sweden, including adaptation of a manualized intervention to the Swedish context (41).

All clinicians overseeing the adaptation process were fluent in both Swedish and English. Adaptations to the EASE manual following the initial clinical workshops were directly translated into Swedish and formulated into the shortened intervention. Given the rapid need to implement this work in response to the increased community violence, a pragmatic decision was made to not conduct a formal translation of the original manual was conducted as part of this work.

### Community workshop

Following the initial clinical adaptation procedures, dialogue between civil society and stakeholders was initiated. An initial workshop took place at an open-invite event in October 2023, at a local gathering place in Järva. Professionals, volunteers and parents of violence affected youth attended the meeting. Intended purposes of the meeting were to identify the needs of the community, how a task-sharing intervention would be received among beneficiaries, and what should be included in an intervention intended to benefit the community. The therapists who led the initial clinical adaptation oversaw the meeting. More than forty participants attended this workshop. Following the workshops, community members were invited to participate and to stay informed throughout the continuation of the development of the new intervention.

After the initial workshop, there was continuous dialogue, both online and in-person with stakeholders in the local area to discuss the ongoing changes. No incentives were given for this engagement. The workshop was organized as an open-invitation community event. As attendance was not formally recorded, the exact composition of participants is unknown.

### Feedback

After the initial adaptation of the material, integrating clinical and community feedback, the adapted manual was sent to therapists employed at Save the Children Sweden with extensive experience working with children exposed to violence, who were not involved in any capacity with the program for feedback (mean practice time: 12 years). They were tasked to provide feedback on the adapted manual, including language, adherence to CBT-skills, and the feasibility of delivering the intervention by task-sharers.

### Refinement of intervention materials with user feedback

Following adaptation, iterative feedback on the intervention materials was obtained from children residing in the community. Feedback was collected at several stages of development. First, individual components of the material (e.g., workbook content and CBT-based exercises developed for related activities within the treatment unit) were reviewed by children from the target community, who provided feedback on content, comprehensibility, and relevance. Second, the complete manual was reviewed in a group review session with four children living in Järva, who provided feedback on the material and its acceptability. The purpose was not to evaluate the intervention or its outcomes, but to inform further refinement of the materials. Finally, prospective group leaders provided feedback on both the content and the feasibility of delivering the intervention. The Bernal Framework was used to organize and guide adaptations (33).

## Results

### Clinical adaptation

To ensure community acceptance of the adapted invention, clinicians suggested shortening the intervention (Table 1). This first step resulted in EASE being shortened from seven sessions for children to four, and three sessions for caregivers to one. There were a number of reasons suggesting this. Firstly, the aim was to the potential of disseminating a method among many children in an area with a high degree of exposure to community violence. To do this with the original version of EASE would have been costly in both operational cost and person-time. Furthermore, Sweden is a country with a well-developed health and medical care system. The purpose of the intervention is not to replace the healthcare system, but to offer a complement, and ultimately a bridge to healthcare. If the intervention had been seven sessions for children and three sessions for caregivers, as in the original version of EASE, it would have been in line with many of the child psychiatric interventions offered by the health system. For families, more sessions in turn may have been misinterpreted as treatment. Additionally, the associated personnel costs and time commitment for two extensive interventions would be difficult for families residing in targeted areas. Secondly, given Swedish legislation on what constitutes health and medical care, EASE in its original form may be considered treatment. Given prior needs assessments conducted, such as those after the high influx of refugees in 2015, it was determined that such efforts are not what is primarily needed, given the availability of more extensive care.

**Table 1:** Example of changes made in accordance with the Bernal framework.

Domain	Implementation	Rationale	Relevant for
Language	Language adapted to more child-friendly and age-appropriate versions	The language was not considered to fit the linguistic context.	Implementation
Persons	Instructions on how the intervention can be implemented with different age groups: *Children (7–12)*: use of toys, changes to the material Youth (13, 14): exercises instead of games, sometimes deepening the material	The material was deemed to need a higher level of interactivity among participants to fit in with similar programs in Sweden. Initially material was deemed to be perceived similar to a school lesson/desk-like teaching.	Training
Metaphor	Remove activities that were not accepted in the cultural context and add more relevant examples: E.g. remove sweeping the floor for the parents	All child psychologists, young people who have been asked and active in the area do not assess that this information is contextually appropriate. Activities that were included instead were: paint with siblings, go to basketball	Adaptation
Content	Included a referral system including training and questionnaires	In this context, children have both the right to care and the right to access treatment.	Training, implementation, supervision
Concept	Rewriting the history that follows the treatment to fit the experiences that are common in the area	The history of EASE was deemed irrelevant for the context and rewritten by therapists. The text was then worked on together with the group leaders working in the community.	Implementation
Goals	Goals in the stories were changed	The current goals were not deemed to fit the Swedish context and were changed	Implementation
Methods	Behavioral activation was removed as a separate session and instead integrated into other sessions.	Behavioral activation was removed as a separate session as the primary problems for the children were not related to inactivity.	Implementation
Context	Training on child safeguarding and Swedish child protective legislation	The group leaders need to have expertise about when we need to report to social services in order to comply with Swedish legislation and also internal guidelines.	Training, implementation

When comparing Sweden to other countries that have evaluated models of task-sharing, differences exist in terms of availability and access to healthcare. In Sweden healthcare is provided free of charge for all children regardless of citizen status. Therefore, there is always a possibility to combine the newly developed implemented method with healthcare, if needed. Models of task-sharing being implemented in low-resourced development or humanitarian settings are often tangential to the healthcare system due to limited access or existing strain.

We also built on lessons learned through previous initiatives (41). A key lesson was that identifying children in need of additional support was often insufficient to ensure that they accessed appropriate services. Many families experienced barriers when navigating the healthcare system, particularly in socioeconomically vulnerable settings. To address this, UNITE incorporated a pilotage component (Swedish: vårdlotsning), involving close collaboration between task-sharers and supervising clinical psychologists.

Throughout the intervention, task-sharers could consult psychologists regarding children who appeared to require additional support based on screening results, observations during sessions, or requests from children and caregivers. When further care was deemed necessary, support extended beyond providing information or formal referrals. Depending on the needs of the child and family, pilotage could include assistance contacting services, coordination between healthcare, school, and social services, support during the referral process, or facilitating initial contact with care providers. The aim was to reduce barriers to care and support access to existing services rather than replace them.

**Figure 2:**
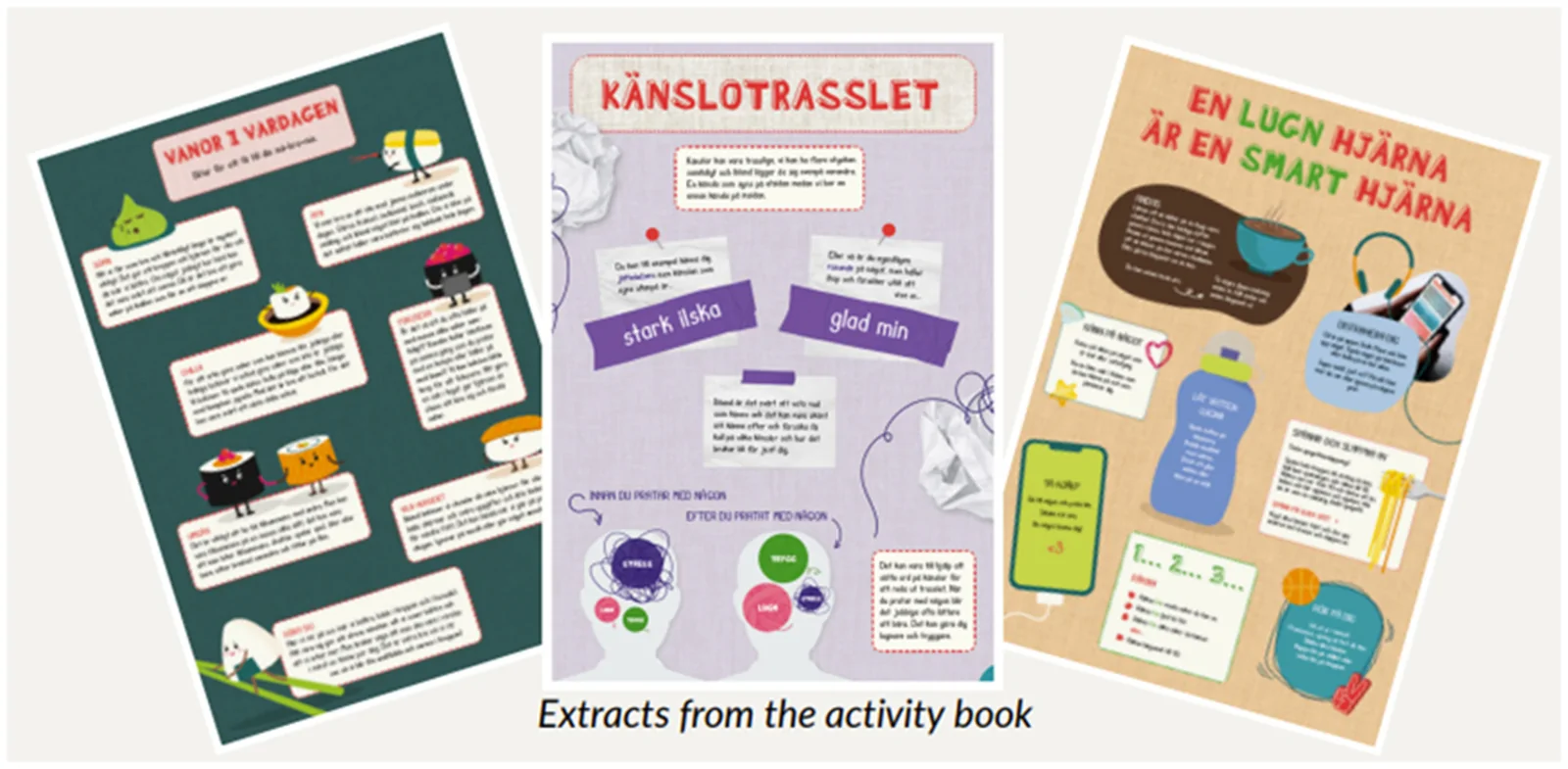
Examples of pages from the UNITE activity book used to support psychoeducation and skills practice throughout the intervention. Adapted with permission from UNITE - Activity Book by Save the Children Sweden. Copyright © Save the Children.

In addition to above mentioned changes, an adaptation around making the sessions and the additional material relevant to children in the Swedish context was conducted. This has been done by including more exercises and games in the material. We have worked together with a layout firm to make material appealing both visually and communicatively. Finally, language that was deemed difficult for children following the initial translation was further simplified. Figure 2 includes examples of how the new manual was illustrated to ensure relevance to children residing in Sweden

### Cultural and contextual adaptations

Several cultural and contextual changes were made, based on the feedback given from the community workshop and the ongoing dialogue with stakeholders. In these communications it became clear that there is a need for interventions specifically related to mental health since many children already participate in other leisure activities that do not have a focus on their mental wellbeing. According to personnel from schools, these measures could be integrated with after-school activities to effectively reach children. It was also clear that it is important to address similar needs among caregivers. It was essential to create opportunities to meet with caregivers and address their worries but also that the intervention needs to be grounded in local ownership and acknowledges that parents may worry about what their children might disclose.

Some stakeholders emphasized the stigma associated with mental health care and highlighted the need to find appropriate language to normalize reactions and offer support with a low-threshold for inclusion. Additionally, the importance of reaching out to children and parents who do not typically participate was emphasized. Finally, suggestions were made to involve children in the development of sessions and to explain various aspects of the Swedish system to them so they can understand and benefit from the available support.

To begin with, the original material contains a story about a child that is read out loud during the sessions. The story needed to be re-written to fit the Swedish context as the examples contained in the original storybook were not necessarily relevant. The story was re-written in two stages. At first, it was rewritten by one of the psychologists and reviewed by two other clinicians and two group leaders. Subsequently, it was piloted with task-sharers who then saw that further adaptation was required and a teenage version was created. It was then determined that two manuals were required for the targeted age ranges: one for children aged 9–12 and another for teenagers aged 13–14. This decision was made to ensure the material would be appropriately received by the older age groups. Table 1 presents examples of clinical, cultural, and contextual changes made, outlined in accordance with the Bernal Framework.

### UNITE - the new intervention

The integration of all materials was done by clinical psychologists based on the feedback acquired in the clinical and community workshops, and the suggested adaptations. After integration, the manual was tested again against pilot groups, young people and group leaders. Subsequently, final feedback was integrated.

The resulting intervention, named UNITE, consists of four sessions of 120 min. The intervention is implemented in groups of 4–8 participants and provided by at least one group leader and one additional adult. The group leaders are trained by psychologists overseeing the implementation of UNITE. The resulting training was 5 days and group leaders were provided weekly supervision throughout implementation.

**Figure 3:**
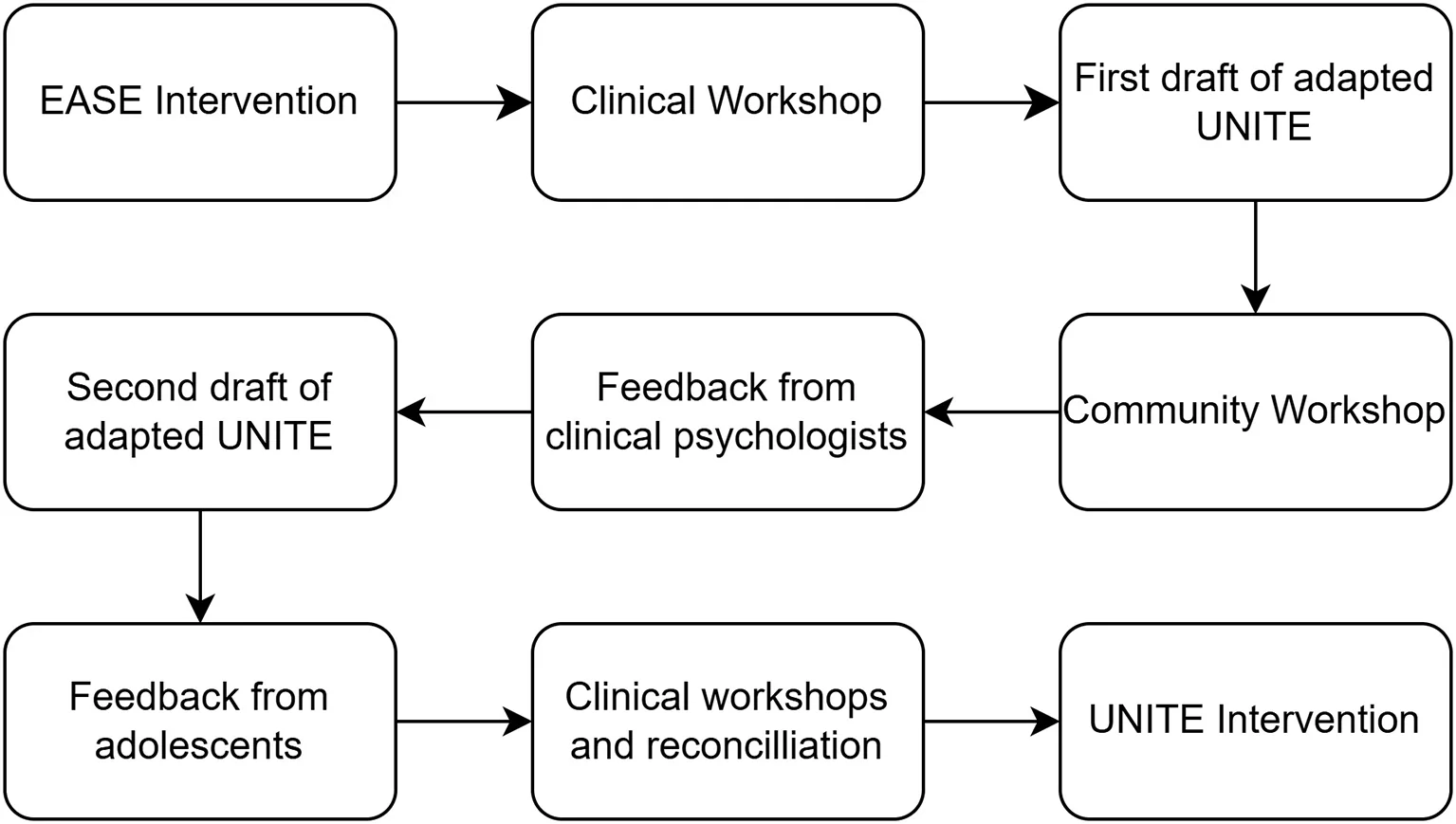
Overview of the adaptation process used to develop the UNITE intervention from the original EASE intervention. The process included clinical workshops, community stakeholder input, iterative revisions, adolescent feedback, and clinical reconciliation.

As part of UNITE, the group leaders initially invited the parents to provide the parental session which also works as an onboarding session. The session primarily consists of psychoeducation. The reason why the parental session is first is twofold. First, it is about ensuring the parents' investment in the child's participation. And secondly, because this was deemed the most pragmatic as the parents' consent is required for children to participate in the intervention. The first session for children consists of psychoeducation, relaxation skills and mentalization. Further, the children get to understand reactions to stressful events, increase understanding of both their own and others feelings, managing stress and skills connected to participating in a group. The second session consists of emotion and affect regulation. This session includes being able to identify and name emotions, find ways to express emotions, handling strong emotions, and being able to properly identify both one's own and others thoughts and emotions. Lastly, the session includes self-care skills. The third session goes into teaching how thoughts and feelings are connected. It continues with ways to handle unhelpful thoughts; provides skills to manage strong emotions and problem solving. Additionally, it repeats skills from former sessions about being able to properly identify and think about both your own and others thoughts and emotions. The fourth session included repetition and knowledge on the Swedish health-care system. The session includes expressing needs, being able to properly identify and think about both your own and other people's thoughts and emotions, and self-care. The rationale is that the children should be better equipped in understanding their own needs and information about where help is available. The final session also includes a reflection on their own wellbeing and a possibility of being connected to further support through the licensed personnel either directly or indirectly. Figure 3 presents an overview of the final UNITE intervention, and how it relates to the original EASE intervention.

## Discussion

There are currently few existing psychological interventions that use task-sharing tailored for children in high-income contexts. Although task-sharing approaches have recently been explored in several high-income countries, particularly among adult populations, evidence regarding child-focused interventions remains limited. This paper outlines a pragmatic approach in adapting an evidence-based scalable psychological intervention, EASE, to the Swedish context, for a rapid response to increased community violence.

Throughout the adaptation, we found that several major adjustments to the materials were needed in the form of scope, appearance and examples. At the same time, it became clear that the main active ingredients and content of EASE could be retained without dramatic changes. The exception was the behavioral activation component, which was not considered to be a primary focus for the population of interest. Sessions for specific work with behavioral activation were hence removed. However, during all sessions, children will partly work with behavioral activation and strategies to enhance their wellbeing.

Due to the increase of community violence, and in turn the rapid need for such a program to be implemented, the adaptation was conducted in a real-world setting over three months. Rather than using Bernal's Framework as an adaptation model, we used it as a framework for reviewing and reflecting on the iterative adaptation process and finalized intervention. This allowed us to assess whether the adaptations developed through stakeholder engagement addressed relevant cultural and contextual dimensions, while maintaining a flexible and pragmatic adaptation process. However, viewed through the lens of Bernal's Framework, several of the final adaptations related to its dimensions such as language, content, methods, and context. Since the adaptation process was highly iterative and part of real-world programming rather than isolated research, adaptations were recorded at a group level as opposed to being linked to individual participants or specific stakeholder groups.

Observational research will take place during implementation, with additional qualitative work to understand the experiences of beneficiaries and implementers. The pragmatic adaptation should not be seen as a shortcoming as it allowed for an evidence-based intervention to be rapidly adapted and implemented as a response to an ongoing national situation. Lessons learned throughout this process, and subsequent implementation research on the application of the program in communities will be useful for similar future initiatives. It should be seen as a pragmatic adaptation, that will have room for refinement as needed in the future.

An observational pilot study of UNITE is currently underway. In the study, we will investigate both feasibility and acceptability of the method in areas characterized by societal violence in Sweden. In addition to this, a qualitative process evaluation will be conducted. The study has been approved by the Ethical Review Board (Dnr: 2023-08194-01 and 2024-05883-02) and began shortly after the final version of UNITE was approved. As part of the pilot study, we will observe a minimum of 60 children getting the intervention, and collect data on mental ill-health, wellbeing and use of health care services. Qualitative research will focus on both childrens, parents and faciliatators experience of receiving and delivering the intervention. In the event of a positive outcome of this work, the intervention will be made publically available via Save the Children's Resource Center.

The ambition of developing UNITE was to adapt an evidence-based intervention to enable rapid deployment in areas affected by community violence. Participation is based on geographic and school-based inclusion rather than symptom thresholds. UNITE is intended to be accessible to children with diverse experiences and levels of need within affected communities. The need to rapidly mobilize available resources in crisis settings is substantial, and interventions often need to be scaled up within weeks, sometimes even days. The ability to adapt existing materials for use in new crises is therefore critical. In this work, we found that it was feasible to adapt previously developed materials to a new context. By systematically documenting this adaptation process, we aim to build on these experiences as additional task-sharing interventions become available.

The adaptation also comes with some limitations. A more systematic adaptation approach, including, for example, cognitive interviews, will be useful in the future to further tailor UNITE for the Swedish context, and other comparable contexts. This is something that we will need to investigate more closely in the future. Furthermore, we are also aware that there are other models that could have been used rather than Bernal's Framework. However, the model had previously been used to adapt similar psychological interventions (34), and it was deemed to be relevant to this with the given conditions that existed. Another limitation is that behavioral activation was removed during the adaptation process, and the implications of this modification for intervention effectiveness remain unknown. Future studies are needed to evaluate the impact of this change.

Finally, we acknowledge that adaptation processes involve balancing multiple perspectives and practical considerations. Feedback from community stakeholders, helpers, and clinicians was collected and reviewed throughout the development process. The adaptation decisions were ultimately made by the psychologists responsible for the intervention, but these decisions were informed by an iterative process in which stakeholder perspectives frequently challenged professional assumptions regarding what would be most appropriate or desirable. In several instances, adaptations were made to improve accessibility and relevance for the target population. At the same time, decisions also needed to consider legal, organizational, and safeguarding requirements related to healthcare services, schools, and social services. We therefore view the final version of UNITE as the product of an ongoing dialogue between participant experiences, professional expertise, and the broader systems within which the intervention is intended to operate.

## Conclusions

Here we describe the development of UNITE, adapted from EASE, to enable rapid implementation in a high-income context. The process showed that relatively large changes were needed in both format and presentation, informed by national legislation and the structure of the healthcare system, while the evidence-based psychological active ingredients remained relevant and could be retained. As such, this study contributes to the limited literature describing the adaptation of scalable psychological interventions from low- and middle-income countries to high-income settings and provides a practical example of how task-sharing principles can be translated to a Swedish context.

In recent years, an increasing number of psychological interventions have been developed to address psychological distress and mental disorders in low- and middle-income countries. At the same time, similar needs have been identified in high-income countries, particularly in the context of increased forced displacement globally. Solutions developed and tested in development and humanitarian settings provide a valuable starting point for addressing unmet mental health needs in high-income contexts. However, adaptations are necessary to ensure cultural acceptability and alignment with existing healthcare systems.

Rather than functioning as stand-alone services, interventions in settings such as Sweden can be integrated alongside healthcare systems to complement existing services and expand access to care. This may contribute to increased service provision for populations facing barriers to mental health care, including refugees and other groups in socioeconomically vulnerable or geographically underserved areas.

## Data Availability

The original contributions presented in the study are included in the article/supplementary material, further inquiries can be directed to the corresponding author.
